# Intracellular Loop 2 Peptides of the Human 5HT1a Receptor are Differential Activators of Gi

**DOI:** 10.1155/2012/490734

**Published:** 2012-05-09

**Authors:** Brian Hall, Carley Squires, Keith K. Parker

**Affiliations:** Department of Biomedical and Pharmaceutical Sciences (MPH I02), Center for Structural and Functional Neuroscience, Skaggs School of Pharmacy, The University of Montana, 32 Campus Drive No. 1552, Missoula, MT 59812-1552, USA

## Abstract

Peptide mimics of intracellular loop 2 (ic2) of the human 5HT1a receptor have been studied with respect to their ability to inhibit agonist binding via interference with receptor-G-protein coupling. These peptides give shallow concentration-effect relationships. Additionally, these peptides have been studied with respect to their ability to trigger the signal transduction system of this Gi-coupled receptor. Two signaling parameters have been quantified: concentration of intracellular cAMP and changes in incorporation into the G protein of a stable analog of GTP. In both cases, peptide mimics near midloop of ic2 actually show agonist activity with efficacy falling off toward both loop termini near TM 3 and TM 4. Previous results have suggested that the loop region near the TM3/ic2 interface is primarily responsible for receptor-G-protein coupling, while the current result emphasizes the mid-ic2 loop region's ability to activate the G protein following initial coupling. A limited number of peptides from the receptor's TM5/ic3 loop vicinity were also studied regarding agonist inhibition and G-protein activation. These peptides provide additional evidence that the human 5HT1a receptor, TM5/ic3 loop region, is involved in both coupling and activation actions. Overall, these results provide further information about potential pharmacological intervention and drug development with respect to the human 5HT1a receptor/G-protein system. Finally, the structural evidence generated here provides testable models pending crystallization and X-ray analysis of the receptor.

## 1. Introduction

Regulation of serotonergic (5-hydroxytryptamine; 5HT) function in animals impacts numerous physiological and pathological processes [1]. 5HT is broadly represented in biological systems as a regulator and modulator via nervous, hormonal, and autacoidal means [2–5]. For example, serotonin [6] has been implicated in the pathophysiology of migraine. This association with migraine is shared with many other factors including adipokines such as leptin; hypothalamic hormones, Orexin A and B (also known appetite regulators as is 5HT); numerous neurotransmitters [7]; autacoids; hormones, and ions like calcium, and magnesium. The range of biological molecules that interact with serotonergic processes suggests that various signaling pathways may be shared, and that the potential for dynamic, collaborative regulation exists. Better understanding of the molecular basis underlying these signaling processes is not only critical to greater fundamental knowledge but to therapeutic development.

Various receptors (R), including the 5HT3R's that are ligand-controlled ion channels, are crucial to these regulatory processes [4]. All other known 5HTR's are structurally different than these ion channels, being serpentine membrane R's [8], coupled (C) to the cells interior by G (GTP binding) proteins (P), which in turn regulate key effectors such as adenylyl cyclase (AC, [9]). These GPCR's share the structural characteristic of 7-transmembrane (7TM) helical segments [10–13]. For many years, the only crystal structure was of rhodopsin, the prototype GPCR, in its interaction with the G-protein transducin [14]. Recently a breakthrough has occurred, with crystallization of the beta-adrenergic receptor (BAR) and publication of X-ray structures [15–18]. This long-awaited event has set the stage for other GPCR. Progressive developments have been demonstrated by crystal structures for the adenosine A2R [19], the CXDR4 chemokine R [20], and the dopamine D3R [21]. Crystal structures for other GPCR, including that those 5HTR's that are GPCR's should soon follow [22].

Of the GPCR recognized as 5HTR, the 5HT1aR (a relative of BAR) is one of the most highly studied [23–25], and it has been associated with physiological and pathological processes as diverse as thermoregulation, cognitive flexibility, and control of mood [26–33]. Depression, underlying anxiety disorders, and related psychopathologies are a particular theme [34–39]. Multiple strategies have been used to dissect the complex pathways underscoring these physiological and pathological processes [40–47]. One approach centers around analysis of allosteric sites of action on receptors. Peptide mimics of intracellular loop regions of 5HT1aR have been used as probes of the receptor-G-protein interface in this context [48–54]. The current communication continues our analysis with these peptide probes particularly emphasizing intracellular loop 2 (ic2) with some, limited comparative data from intracellular loop 3 (ic3). The results with ic2 and ic3 are suggestive of potential sites for regulation and therapeutic drug development.

## 2. Materials and Methods

### 2.1. Cell Culture

Chinese Hamster ovary (CHO) cells expressing the H5-HT1aR [55, 56] were cultured in Ham's F-12 medium fortified with 10% fetal calf serum and 200 ug/mL geneticin. Cultures were maintained at 37°C in a humidified atmosphere of 5% CO_2_. Cells were subcultured or assayed upon confluency (5–8 days). Cloned H5-HT1aR was kindly provided by Dr. John Raymond (Medical U. of South Carolina; [41]). The cell line has been tested for mycoplasma with a PCR kit (ATCC) and is free of contamination.

### 2.2. Receptor Preparation

Cells were trypsinized and centrifuged at low speed in ice-cold medium [53]. The pellet was resuspended in ice-cold Earle's Balanced Salt Solution followed by centrifugation. Cells were resuspended in 10 mL of ice-cold binding buffer (50 mM Tris, 4 mM CaCl_2_, 10 *μ*M pargyline, and pH 7.4), homogenized with Teflon-glass, and centrifuged for 450,000 g-min. at 4°C. For a crude membrane preparation, the pellet was resuspended in 30 mL of ice-cold binding buffer, homogenized on Teflon-glass and then by Polytron (setting 4) for 5 seconds, and stored on ice and assayed within the next 1.5 hours [54].

### 2.3. Assay of Receptor Activity

Binding of the agonist [3H]8-OH-DPAT ([3H]8-hydroxy-2-(di-n-propylamino)tetralin) to H5-HT1aR followed well-characterized protocols [49, 50, 53]. Radioligands were purchased from New England Nuclear (NEN), Boston, MA, and 1 mL reaction mixtures, in triplicate, were incubated for 30 min. in a 30°C shaker. The 1 mL mixture was 700 *μ*L of receptor preparation; 100 *μ*L of binding buffer (for total binding) or 10 *μ*M 5-HT (for nonspecific binding), 100 *μ*L of the tritiated agent (concentration of 0.5 nM [3H] 8-OH-DPAT), and 100 *μ*L of peptide or binding buffer in the case of controls.

Reactions were stopped by addition of 4 mL of ice-cold 50 mM Tris buffer, pH 7.4, and vacuum filtration on glass fiber filters (Whatman GF/B). Filters were rinsed twice in 5 mL of ice-cold Tris buffer, dried, and counted in 5 mL of Ecoscint (National Diagnostics) liquid scintillation fluid in a Beckman LS 6500. Homogenates were assayed for protein to maintain a nominal value of 50 *μ*g protein per filter [57]. All tubes were run in triplicate.

### 2.4. cAMP Assay

 CHO cells were cultured to confluency in 12- or 24-well plates. Medium was aspirated, and the cells were rinsed twice in warm, serum-free F-12 medium. Cells were incubated for 20 min. at 37°C in 0.5 mls of serum-free F-12 medium containing 100 uM isobutylmethylxanthine (IBMX) and the following substances (final concs.) alone or in combination (see Figures 3 and 5): 30 *μ*M forskolin (FSK; for all treatments); 1 *μ*M 5-HT; peptide concentrations as noted in figure legends. Reactions were stopped by aspiration of medium and addition of 0.5 mL of 100 mM HCl. After 10 min., well contents were removed and centrifuged at 4000 rpm. Supernatants were diluted in 100 mM HCl, and cAMP was quantified [53] directly in a microplate format by enzyme immunoassay (EIA) with a kit from Assay Designs (Ann Arbor). Triplicate-independent samples were assayed.

### 2.5. [35S]GTP*γ*S Assay

 H5-HT1aR membranes from transfected CHO cells were incubated with 5-HT (0.1 *μ*M) and/or peptide concentrations as noted in figure legends (see Figures 2 and 4) and the following incubation mixture: 20 mM HEPES buffer, pH 7.4, 5 mM MgCl_2_, 1 mM EDTA, 1 mM DTT, 100 mM NaCl, 100 uM GDP, 10 *μ*M pargyline, 0.2 mM ascorbate, and 0.1 nM [35S]GTP*γ*S [53, 58]. Mixtures were incubated for 30 min. at 30°C, and were terminated by dilution in cold buffer. The mixture was filtered on GF/C filters, rinsed twice in buffer, dried and counted by liquid scintillation. All values reported in are for specific binding (total nonspecific) of triplicates. Nonspecific binding was determined in the presence of cold *γ*-S-GTP (10 uM). Negative control is the above mixture minus test drug or 5HT. Positive control contains 5HT.

### 2.6. Data Analysis

All statistics (means, standard errors of the mean (SEM), *t* tests and ANOVA, Pearson correlation coefficients (*r*), and graphical procedures (including drug-receptor-binding analysis) were conducted with PSI-Plot (Version 8) software (Poly Software International), Prism (version 4.0c), or using a Hewlett-Packard Graphing Calculator, HP48. The apriori was *α* = 0.05 for all experiments. Experiments were conducted with a minimum of *n* = 3, in triplicate. Most experiments were *n* = 3–5. In some cases (indicated in figure legends), different *n*'s and multiplicates were used.

### 2.7. Peptide Preparation

These highly purified (greater than 95%) peptides were purchased from New England Peptide LLC. The peptides are segments of ic2 and ic3 of the cloned H5HT1aR. Peptides stored at −20°C were initially dissolved in deionized water. Subsequent dilutions were in binding buffer. The peptides examined in this study are listed in Table 1.

## 3. Results

### 3.1. Intracellular Loop 2 (ic2)

 The size of H5HT1a's ic2 (about 20 amino acids) makes it a tempting target for analyzing the loop's coupling to and activation of Gi [23, 24]. Our previous work with ic2 emphasized the N-terminal region of the loop with a peptide we call P11 (Table 1). Results with this peptide suggest that the loop residues near TM 3 are vital for coupling of the loop to Gi but are not involved in G-protein activation [53]. Results from the Varrault group [48] looked at the entire loop without distinguishing subregions; their conclusions were that the entire loop is responsible for activation (they did not differentiate between coupling and activation). The following question arises: can coupling and activation characteristics be identified for the loop on a subregional basis? Our preliminary work at the N-terminal aspect of H5HT1a'a ic3 suggested to us that the techniques we use could be productive in addressing such a question [49, 50, 53]. Thus, we synthesized peptides of 10 residues each that progress from the N-terminus of ic2 to the C-terminus two amino acids at a time (Table 1). Beyond the parent peptide, P11, this results in seven additional peptides (P21–P27). Agonist inhibition [59, 60] was used as a measure of coupling efficacy. Any agent or process that uncouples a receptor from its G-protein partner increases the probability that the receptor will be in a lower affinity state for agonist binding. This results in concentration-dependent agonist dissociation relationships that reflect affinity of the uncoupler for the G protein (and potentially by analogy the affinity of the cognate receptor loop region for the G protein). Two determinants of G-protein activation (stable GTP binding to Gi and changes in intracellular cAMP concentration) were used to monitor a peptide's ability to perturb G protein following coupling. The overall results for these eight peptides are in Table 2.

**Figure 1:**
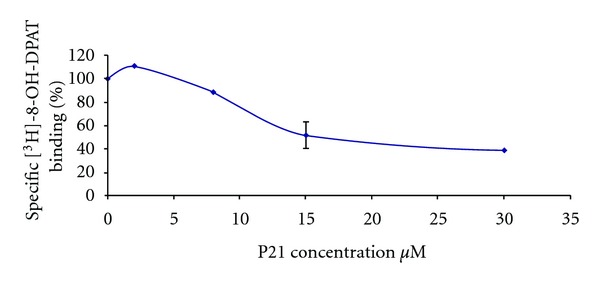
P21 noncentration-dependent displacement of bound 8-OH-DPAT. This curve represents the change in specific binding of [^3^H]-8-OH-DPAT, a 5HT1aR agonist, to the receptor in the presence of various concentrations of the ic2 peptide mimic P21. Nominal binding of agonist at control levels was 400 fmoles/mg protein.

As shown in Figure 1 with results from peptide P21 as an example, these peptides give shallow concentration-effect curves for the measure of coupling and agonist inhibition. Similar experiments with all peptides form the basis for the summarized coupling results found in Table 2. Note that limited peptide solubility and lack of efficacy prevented complete IC50 determination for all peptides (P24–27). The uM concentration ranges for activity of these peptides, and, shallow concentration-effect relationships in, are typical for other peptides we and others have analyzed [48, 53].

**Figure 2:**
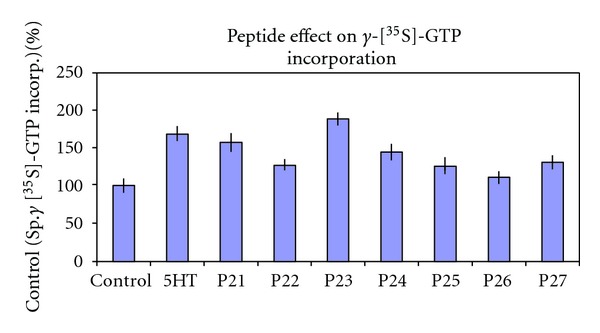
ic2 peptide effect on *γ*-[^35^S]-GTP incorporated into Gi, a measure of G-protein activation. Control is the basal amount of *γ*-[^35^S]-GTP incorporated into Gi in CHO cells expressing the human 5HT1aR, set as 100%. The *Y*-axis is the percent of specifically bound (total minus nonspecific) *γ*-[^35^S]-GTP. All other treatments are percents of the control value. All peptides are 30 uM concentration and 5HT 10^−7^ M concentration. Error is expressed as SEM.

**Figure 3:**
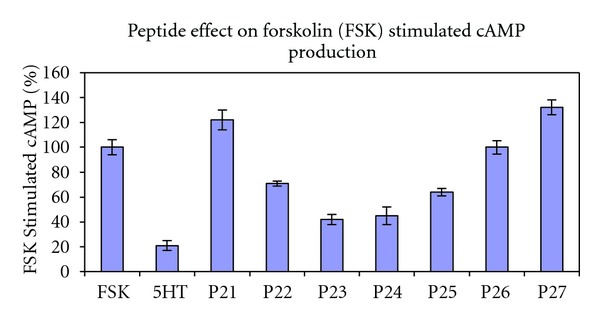
ic2 peptide effect on forskolin-stimulated cAMP production, a measure of activated-G-protein regulation of adenylyl cyclase. Forskolin (FSK) stimulated cAMP production by adenylyl cyclase (AC) is in CHO cells expressing the human 5HT1aR. FSK (30 uM) is the control, which is set to 100%. All other treatments are expressed as a percent of the control value. Peptide concentrations are 30 uM. All treatments include isobutylmethylxanthine (IBMX) an inhibitor of the metabolism of cAMP by phosphodiesterase. Error is expressed as SEM.

Figure 2 gives results for the eight peptides' ability to foster incorporation of GTP into Gi using a radioactively labeled, reasonably stable form of GTP ([35S] gamma-S-GTP). Relative to control (buffer alone; no agonist nor peptide) midloop residues as represented by peptide P23 are most effective in directing incorporation of GTP into Gi. Efficacy declines in both N- and C-terminal directions from P23 although the results for P21 are anomalous in this regard. It is not clear whether this result for P21 is meaningful or due to experimental error although the results for intracellular cAMP (Figure 3) may shed some light on this situation. Note that GTP binding by Gi is an agonist-dependent process (see 5HT in the Figure); thus, when peptides increase GTP incorporation above control level, the implication is that the peptides are representing native loop regions under the influence of agonist. 

Figure 3 shows a parallel set of results whereby the peptides' ability to change intracellular cAMP concentrations following coupling to Gi is determined (control is the FSK stimulated level; agonist; e.g., 5HT activates Gi and lowers cAMP levels below the control reading). Again, peptide P23, representing mid-loop residues, is most effective. In contrast to the results for GTP incorporation in Figure 2, the cAMP results have a smooth drop off in efficacy on both sides of P23. Overall, the trends peaking at P23 and declining on both sides are parallel for GTP incorporation (Figure 2) and cAMP concentrations (Figure 3). Note that basal levels of intracellular cAMP are quite low, and the experimental protocol for these experiments involves artificially raising cAMP concentrations via stimulation of adenylyl cyclase with forskolin (control) and comparison of peptide results to that produced by the agonist serotonin. 

### 3.2. Intracellular Loop 3 (ic3)

**Figure 4:**
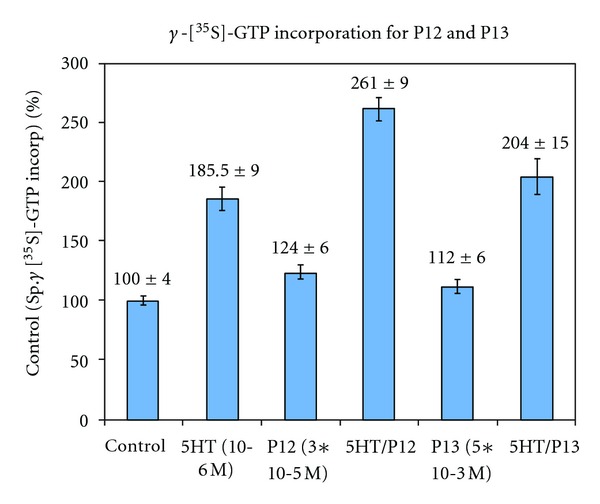
P12 and P13-stimulated incorporation of *γ*-[^35^S]-GTP control is the basal amount of *γ*-[^35^S]-GTP incorporated into Gi in CHO cells expressing the human 5HT1aR set as 100%. The *Y* axis is the percent of specifically bound *γ*-[^35^S]-GTP. All other treatments are percents of the control value. Peptide concentrations are 30 uM. *P < 0.01 P12 versus control; ^*€*^ *P* < 0.01 5HT versus 5HT/P12. *P13 versus control *P* < 0.01; ^*€*^5HT versus 5HT/P13 *P* < 0.01.

**Figure 5:**
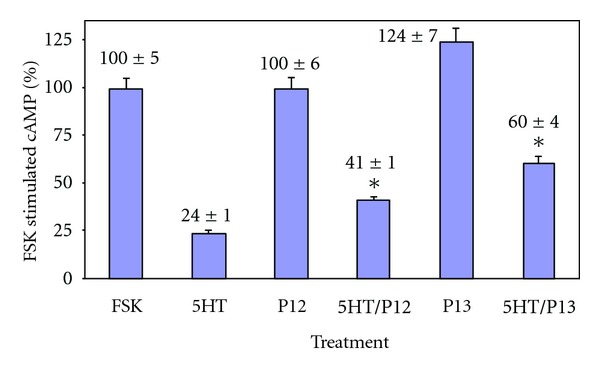
P12 and P13 effect of forskolin-stimulated cAMP production forskolin (FSK) stimulated cAMP production by adenylyl cyclase (AC) in CHO cells expressing the human 5HT1aR. These experiments were a measure of second messenger regulation by G protein. FSK is the control, which is set to 100%. All other treatments are expressed as a percent of the control value. Peptide concentrations are 30 uM. All treatments include isobutylmethyl xanthine (IBMX), an inhibitor of the metabolism of cAMP. 5HT versus 5HT/P12 and 5HT versus 5HT/P13 *P < 0.05.

H5HT1aR's ic3 is much larger than ic2 (about 130 amino acids); nevertheless, we did a very limited number of comparisons at the N-terminal (TM5) region of ic3, continuing preliminary work [50–53] and using the same approach as with ic2 by synthetically building 10-MER's two amino acids at a time from the parent (P1; Table 1).  Table 3 gives coupling and activation summaries for the two peptides, P12 and P13 (Table 1). As with the ic2 peptides, the ic3 peptides, P12 and P13, give shallow, uM concentration-effect relationships (data not shown in graphical form as in Figure 1). For coupling, if 50% is listed, then that is the IC 50; if another value is listed, that is the maximum inhibition possible with the highest soluble concentration. Both P12 and P13 produce small but significant incorporation increases of GTP based upon the amount of [^35^S]-GTP incorporated into Gi (Figure 4), while the outcomes for changes in intracellular cAMP concentrations are more complex (Figure 5): for peptide P12, intracellular cAMP concentration is not altered; unusually, peptide P13 actually increases intracellular cAMP concentration. A possible explanation for the combined results for P12 and P13 is given in the Discussion section.

## 4. Discussion

H5HT1aR is linked to numerous important physiological and pathological processes. Additionally, the receptor is a close relative, not only of other 5HT1 type receptors, but also the beta adrenergic receptors and other GPCR's [13, 42, 55, 56]. Because of these characteristics, structural determinations of the receptor are crucial matters. Despite recent critical structural advances with the beta adrenergic receptors [10, 15, 16, 18], the 5HT1aR is uncrystallized and its structure awaits X-ray analysis [22].

In previous work [49, 50, 53, 54], we have demonstrated the utility of an agonist-based inhibition system associated with signal transduction parameters to study interactions of the receptor with its cognate G protein, Gi. In the current investigation we have presented further information about the H5HT1aR/Gi interface that should provide testable hypotheses anticipating the ultimate structural analysis of the receptor.

Data collected in previous and current experiments have implicated a role for ic2 in receptor coupling and G-protein activation. The N-terminus end of ic2, involving the sequence IALDRYWAITDPIDYV and including peptides P11 (previous work) and P21–P23 (current work), is important for coupling to the G protein. Evidence for this includes presence of the highly conserved DRY sequence for GPCR's [51] and from the present study, IC50's for the peptides' coupling capacity, with ranges from 7 to 30 uM. Decay of G-protein coupling activity was observed as the peptides progress towards the C-terminus of ic2 (P24–27). As the amino acids seem to wane in importance for receptor coupling, they increase for part of the distance in importance for G-protein activation with its peak at the P23 amino acid stretch WAITDPIDYV. This is clearly shown by the bell-shaped progression of the data bars for the incorporation of *γ*-[^35^S]-GTP into Gi_*α*_ (Figure 2). This can be superimposed over the inverted bell-shaped depression for intracellular levels of FSK-stimulated cAMP production (Figure 3) following peptide treatment.

The C-terminal end of ic2 consisting of the amino acids RTPRPR may serve as an anchor, helping to hold the amino terminal of ic2 in a favorable orientation for coupling to the G protein [48]. Also interesting about the carboxy terminal end of ic2 is the presence of the 2 proline (P) residues separated by only 1 amino acid. These proline residues in close proximity to each other introduce a kink in the receptor structure constraining its range of motion. These data demonstrates the clear role for H5HT1aR's ic2 in coupling receptor to G protein, and toward the loop's middle, G-protein activation.

For ic3, the inhibition of AC by Gi is an important regulator of intracellular signal transduction. The current peptides tested, P12 and P13, differed in their ability to regulate this step in the cascade. P12 was unable to decrease the FSK-stimulated levels of cAMP (Figure 5). This is in contrast to the action of 5HT which was able to significantly decrease the FSK-stimulated levels of cAMP. P13 had the opposite effect; it increased cAMP concentrations (Figure 5)! This suggests that the two new amino acids (AD added to form P13) from ic3 are potentially at the beginning of a region of the loop which has negative regulatory properties on Gi blunting its normal ability to regulate AC. It is interesting to speculate about the differences in data from the *γ*-[^35^S]-GTP (Figure 4) incorporation assays and cAMP assays (Figure 5). P12 slightly increases GTP incorporation while P13 statistically does not. Thus, P12 activates Gi but cAMP changes do not ensue. P13 does not activate Gi, but a cAMP change occurs in the atypical direction. With the relatively small changes produced by these two peptides in both signaling measures, one possibility is experimental error that has not been accounted for. It is possible that the peptides are acting at some sites other than the proposed receptor-G-protein interface or that the process at the interface is more complex.

 The most tantalizing possibility is that the newly explored region represented by P12 and P13 is the beginning of a region of ic3 involved in coupling of receptor to G protein still capable of regulating Gi. Additionally, the perturbation of Gi in this case involves different conformational changes that activate Gi but in a novel way. This would produce the opposite effect on cAMP concentration and would be equivalent to the downstream actions of an inverse agonist at the ligand binding site. Since 5HT1aR is capable of constitutive activity [25], inverse agonism is possible, and it will be fascinating to see if the P12/P13 region is involved in this activity once the crystal structure is available. In this context then, P12 would represent a transitional region between “normal” and “atypical” Gi regulation while P13 is in the atypical subregion.

While the data support this region's (P12/P13) role in receptor-G-protein coupling, the peptides' ability to uncouple declines relative to previously studied peptides whose structures represent segments closer to ic3's N-terminus. P12 and 13 are beyond (toward the C-terminus) the key RFRI region of P1 previously identified as key to that part of 5HT1aR's ic3-N-terminus responsible for G-protein activation [50].

Varrault et al. [48] demonstrated that the C-terminal section of i3 is involved in G protein coupling and regulation. So, if our work can be interpreted to mean that peak coupling and activating properties are associated with ic3's N-terminal residues and Varrault's work can be interpreted to mean that peak coupling and activating properties are associated with ic3's C-terminal residues, then what role will hold for the vast internal region of the loop in 5HT1aR? GPCR ic3's are variable in size in rhodopsin versus 5HT1aR and BAR's, which have larger ic3 loops (at least twice the size of rhodopsin's ic3). It would be meaningful to extend this peptide approach into the midloop region of H5HT1a's ic3, and then as a crystal structure becomes available the comparisons of 5HT1aR loop function with BAR and rhodopsin will be fascinating.

Neither of the peptides (P12 and P13) are as potent as 5HT at incorporating GTP into Gi_*α*_. It is possible that multiple regions are responsible for G-protein activation, and the individual peptides mimic only part of this structure [61], thereby producing a diminished effect relative to 5HT. Also, a given peptide region, even one that is absolutely critical in the native structure, may not have the most efficacious tertiary structure without the full loop being present. It is crucial to point out that the parent ic3 peptide (P1) contains the full TVKK sequence at its N-terminus. This sequence is part of the so-called Ric-8 [62, 63] region that has been shown in other GPCR as crucial to G-protein regulation. Significantly, the P1 relatives (P12 and P13) under discussion in this communication are at a transition point for this sequence; P12 contains the full TVKK stretch while P13 has lost the T! One additional observation may be pertinent. For GTP incorporation, for both peptides, the combination of peptide plus 5HT produces markedly greater incorporation than that produced by 5HT alone. This may suggest that 5HT and the peptides may be perturbing separate sites on the receptor and/or G protein.

In summary, this peptide mimic study for intracellular loops 2 and 3 of the H5HT1aR was designed to examine which segments were involved in coupling and activation of Gi. The results reported here in combination with previously reported work conclude that the amino terminal ends of ic2 and ic3 are important for coupling the receptor and G protein. The activation of G-protein peaks at P23 (WAITDPIDYV) in ic2 (mid-loop). The activity is decreased as the structures move in either direction away from this core sequence. The curious results of increased cAMP concentrations caused by P13 suggests that the two new amino acids (AD) in P13 are the beginning of a new region of ic3 which has negative regulatory properties on Gi. That is, the new region may be one that is not normally activated by agonists; however, in the presence of inverse agonists and the different conformational changes they produce, the new region may couple to and activate Gi in a way that regulates AC in a way we define as inverse agonism. The combined results with H5HT1aR ic2 and ic3 peptides should lead to testable crystallographic hypotheses with drugs having differential intrinsic activities. Beyond the final judgment of these peptide probes in the structural sense, the information produced may be useful as independent pharmacological observations. Pragmatic implications of the work may be relevant in a framework where the multiple, differential activities of the peptides can be used by medicinal chemists to build unique pharmacological agents targeting unutilized sites at the receptor-G-protein interface.

## Figures and Tables

**Table 1:** ic2 and ic3 peptide mimics. The primary amino acid sequences for the H5HT1aR ic2 loop peptide mimics P11 and P's 21–27, and for ic3 (P1, P12, and P13). The receptor's amino terminal is to the left. Sequences for H5HT1aR from Kobilka et al., 1987 [56]. P11 is from a previous study by Thiagaraj et al., 2007 [53], and P1 from Hayataka et al., 1988 [49] (both included for comparative purposes).

P11	IALDRYWAITD
P21	LDRYWAITD**P**
P22	RYWAITDP**ID**
P23	WAITDPID**YV**
P24	ITDPIDYV**NK**
P25	DPIDYVNK**RT**
P26	IDYVNKRT**PR**
P27	YVNKRTPR**PR**
P1	IFRAARFRIRKTVKK
P12	KTVKKVEKTG
P13	VKKVEKTGAD

**Table 2:** ic2 Peptide mimic effect on [3H]8-OH-DPAT binding. All binding inhibition values are percent of control agonist (ag.) bound. The upper portion of the table is for peptides nearer the C-terminus, including P11 from Thiagaraj et al., 2007 [53]. These peptides decreased the specific high affinity binding of 5HT1aR agonist [^3^H]-8-OH-DPAT by 50% at the given concentration. The lower portion of the table (P24 on) is the ic2 peptides toward the C terminus. These peptides were less effective at decreasing specific high affinity binding of [^3^H]-8-OH-DPAT, and values given are percent of control at the given concentration. Values for intracellular cAMP are relative to FSK-stimulated control. All values for incorporation of *γ*-[^35^S]-GTP into Gi are percent of control. Nominal values for control binding were 400 fmoles/mg protein.

Peptide	Conc. (uM)	% cont. ag, bound, (SEM)	[cAMP] (SEM)	GTP Incorp. (SEM)
Control			100 (6)	100 (7)
5HT			21 (4)	168 (12)
P11	7	50 (1)	87 (8)	100 (3)
P21	15	52 (4)	122 (8)	158 (11)
P22	16	51 (2)	71 (2)	128 (9)
P23	30	50 (22)	42 (4)	188 (10)
P24	10	94 (9)	45 (7)	146 (17)
P25	30	87 (12)	64 (3)	126 (10)
P26	30	75 (19)	100 (5)	111 (9)
P27	30	95 (5)	132 (6)	130 (7)

**Table 3:** ic3 Peptide mimic coupling and signal transduction data. Summary of data generated for all ic3 experiments with P12 and P13. P1 is included as a reference, from Hayataka et al., 1998 [49]. Nominal values for control agonist binding were 400 fmoles/mg protein.

Peptide	Agonist (%) inhibition	[35S]-*γ*-S-GTP incorporation% above conro	% Inhibition of FSK-stimulated cAMP
P1*	50 (3 uM)	30 (1 uM)	10 (10 uM)
P12	28 (30 uM)	24 (30 uM)	0 (30 uM)
P13	50 (15 uM)	12 (30 uM)	−24 (30 uM)
